# Dust Storm Fallout: Tiniest Travelers Pose Greatest Infection Threat

**DOI:** 10.1289/ehp.116-a128b

**Published:** 2008-03

**Authors:** Angela Spivey

Dust isn’t just a nuisance—its ability to clog human airways and carry pathogens poses a human health problem. In the sub-Saharan region of Africa, the World Health Organization pinpointed dust storms exacerbated by the dry season and drought as a cause of outbreaks of meningococcal meningitis. Dust from Saharan storms can reach as far away as Florida, with particles smaller than 2.5 μm traveling the greatest distances. Studies have shown that human exposure to these tiny particles is associated with human mortality, and new research now shows that such particles are also more likely to carry health-threatening bacteria **[*EHP* 116:292–296; Polymenakou et al.]**.

During a strong Saharan dust storm in 2006, researchers collected air samples in Heraklion, Crete, an area that often feels the effects of such storms. Particles from the samples were separated according to size (> 7.9 μm, 3.3–7.9 μm, 1.6–3.3 μm, 1.0–1.6 μm, 0.55–1.0 μm, or <0.55 μm) using a machine called a high-volume cascade impactor. The researchers characterized the bacteria traveling on particles of various sizes by taking samples from the machine’s filters, then cloning and sequencing a strand of DNA commonly used to identify bacteria. This process created an inventory of the bacterial gene sequences present in each of the six particle-size ranges. The creation of large clone libraries allows investigators to detect bacteria that are missed using culture methods, as some bacteria are difficult to grow.

The researchers identified clones that were genetically related to pathogens linked to human diseases such as pneumonia, meningitis, and bacteremia, or to pathogens suspected of inducing infections such as endocarditis. Of the sequenced clones related to bacteria that are dangerous to humans, almost half (43%) were found at particle sizes less than 3.3 μm. Spore-forming bacteria such as *Firmicutes* dominated the particle sizes larger than 3.3 μm. Most of these bacteria are nonpathogenic.

The authors conclude that the prevalence of breathable bacteria on small dust particles may pose a significant, widespread health risk to humans, given that dust, especially the smallest particles, is known to travel across continents. To determine just how much of a threat these tiny travelers pose, long-term studies are needed to further investigate how pathogens are distributed across dust particle size, and the distances these pathogens can travel and still survive.

## Figures and Tables

**Figure:**
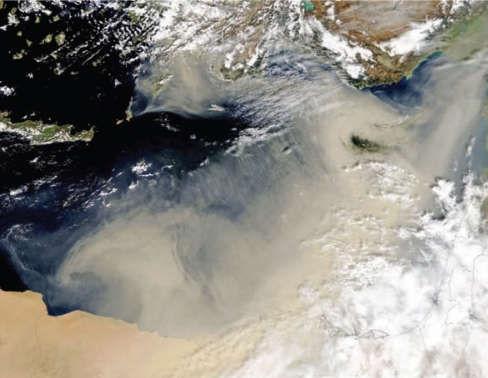
The dust storm that provided samples for Polymenakou et al. obscures the eastern Mediterranean Sea, 25 February 2006

